# CircRNA_036186 mediates HNSCC progression by regulating 14-3-3ζ

**DOI:** 10.3389/fonc.2024.1498139

**Published:** 2024-12-03

**Authors:** Juan Tang, Donglin Yu, Jiaojiao Song, Junfei Li, Yijuan Zhang, Xiangrui Ma, Wenlong Wang

**Affiliations:** ^1^ Department of Oral and Maxillofacial Surgery, Binzhou Medical University Hospital, Binzhou, China; ^2^ School of Stomatology, Binzhou Medical University, Yantai, China; ^3^ Plantation Center, Weihai Stomatological Hospital, Weihai, China; ^4^ Department of Anesthesiology, Binzhou Medical University Hospital, Binzhou, China

**Keywords:** head and neck squamous cell carcinoma, bioinformatics analysis, high throughput sequencing technology, CircRNA, MiRNA, 14-3-3ζ

## Abstract

**Introduction:**

Head and neck squamous cell carcinoma (HNSCC) is a prevalent and lethal malignancy, accounting for 95% of head and neck cancers. Tyrosine 3-monooxygenase/tryptophan 5-monooxygenase activating protein ZETA (14-3-3ζ) is central to various signalling pathways and is pivotal in tumour progression.

**Methods:**

Cancerous and corresponding non-cancerous tissue samples were collected from five patients diagnosed with HNSCC. circRNA and mRNA expression profiles were analyzed using high-throughput sequencing techniques. Potential circRNA-microRNA (miRNA)-mRNA interactions were predicted using bioinformatics tools.

**Results:**

The study found that CircRNA_036186 regulates the expression of 14-3-3ζ in HNSCC through miR-193b-5p.

**Discussion:**

These findings suggest that CircRNA_036186 has the potential to be a biomarker and therapeutic target for HNSCC and provide some theoretical basis for further research on the role of circRNA in HNSCC.

## Introduction

Head and neck squamous cell carcinoma (HNSCC) is a prevalent and lethal malignancy, accounting for 95% of head and neck cancers. The presence of numerous essential structures, including muscles, bones, blood vessels, and nerves, in the head and neck area presents a significant challenge to eradicating the disease using conventional methods, such as surgery, chemotherapy, and radiotherapy. Patients with HNSCC experience a notable decline in their quality of life and mental health (1, 2). Consequently, identifying novel potential biomarkers is paramount for diagnosing, treating, and predicting outcomes in patients with HNSCC.

The 14-3-3 protein is a highly conserved family of molecules. Tissues demonstrate independent subtype-specific functions with seven distinct subtypes (β, γ, ϵ, η, σ, θ, and ζ), each of which is localised in tissues with independent subtype-specific functions (3). The tyrosine 3-monooxygenase/tryptophan 5-monooxygenase activating protein ZETA (14-3-3ζ or YWHAZ) is a member of the 14-3-3 family of proteins, which are central to a multitude of signalling pathways and play a pivotal role in tumour progression (4). The 14-3-3 family interacts with various cellular signalling proteins by binding an amphipathic helix, which is then activated through phosphorylation modifications. Bisphosphorylated polypeptides bind simultaneously and with high affinity at adjacent 14-3-3 sites, forming bidentate complexes with high stability (5).

MicroRNA (miRNA) is an endogenous short non-coding RNA (ncRNA) with a length of 21-24 nucleotides (6). A growing body of evidence from multiple studies has linked aberrant miRNA regulation to the development and progression of various types of cancer (7).

Circular RNA (circRNA) is a pervasively occurring and heterogeneous covalently closed circular endogenous ncRNA (8). The various forms of circRNA include exon circRNA, intron circRNA, and exon-intron circRNA. Exon circRNA is the most prevalent and is predominantly located in the cytoplasm (9). CircRNA has the potential to impede miRNA binding to its target on mRNA, which can influence the expression of downstream target proteins and subsequently enhance the expression of certain genes (10). Currently, circRNA research is primarily focused on exon circRNA. The interaction between circRNA and cancer-related miRNA suggests that circRNA may serve as a crucial regulator in cancer pathogenesis. The occurrence and development of HNSCC concerning circRNA remain incompletely understood.

This study aimed to elucidate the role of circRNA in HNSCC. To this end, software such as TargetScan was employed to analyse the differential expression patterns of circRNA and mRNA in HNSCC and corresponding adjacent samples. Furthermore, the role of 14-3-3ζ in HNSCC was investigated.

## Results

### 14-3-3ζ is highly expressed in both HNSCC tissues and cell lines

**Figure 1:**
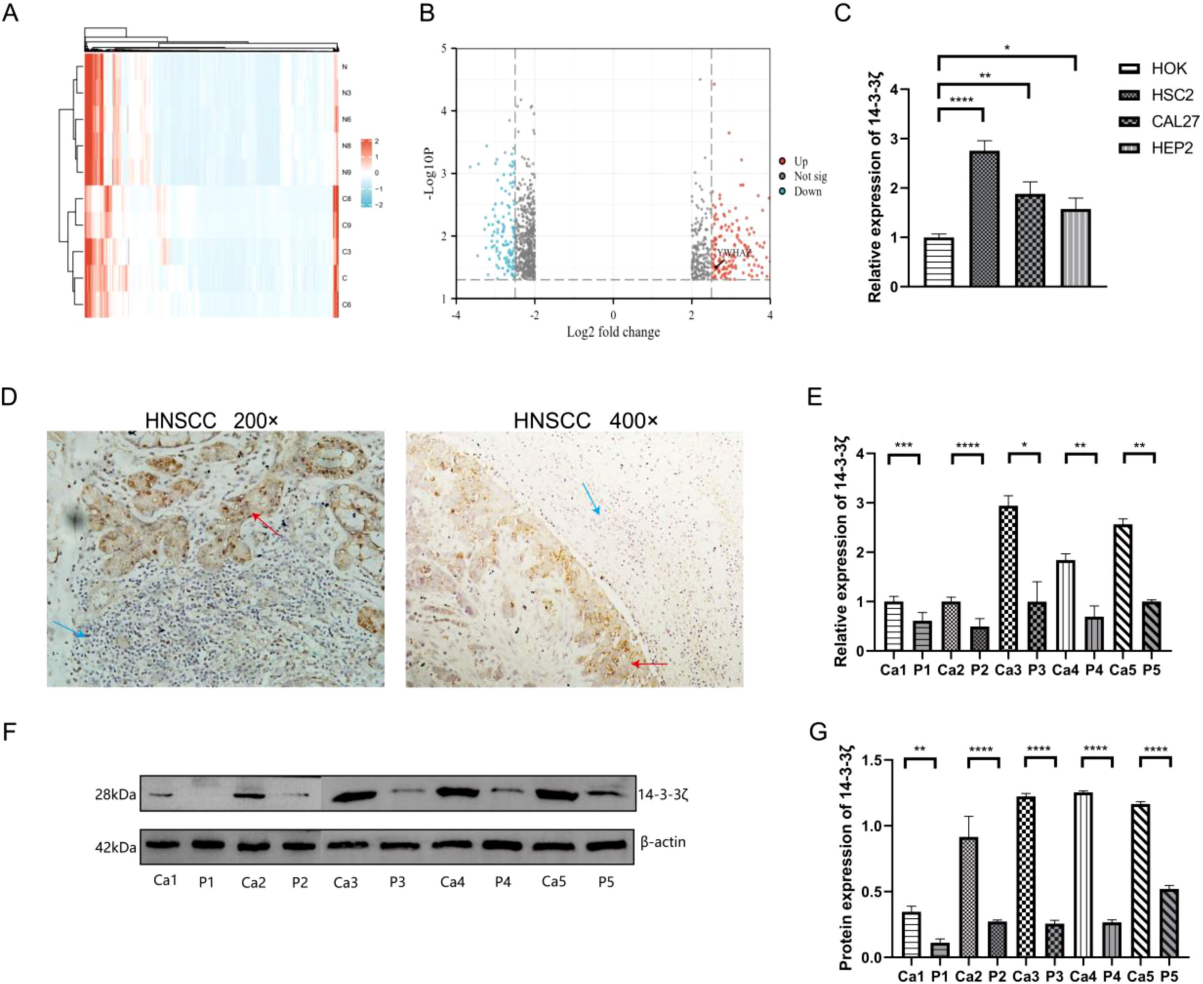
14-3-3ζ is highly expressed in HNSCC tissues and cell lines. **(A)** The heatmap illustrates the expression of 1,053 differentially expressed mRNAs, of which 377 are upregulated and 676 are downregulated. The colouration gradually transitions from blue to red, indicating increased expression. **(B)** Volcano plot for the visualisation of the screened differentially expressed mRNAs. **(C)** RT-PCR was conducted to ascertain the expression of 14-3-3ζ in HNSCC cell lines. **(D)** Immunohistochemistry was employed to detect the expression of 14-3-3ζ in HNSCC tissues. Red arrows, 14-3-3ζ positive, Blue arrows: 14-3-3ζ negative. **(E)** RT-PCR was employed to detect the expression of 14-3-3ζ in HNSCC tissues. Ca, cancerous tissue; P, paracancerous tissue. **(F, G)** Western blotting was employed to detect the expression of 14-3-3ζ in HNSCC tissues. Ca, cancerous tissue; P, paracancerous tissue. Ca, cancerous tissue; P, paracancerous tissue. (n=3, *p<0.05, **p<0.01, ***p<0.001, ****p<0.0001).

A high-throughput microarray analysis identified 35,252 mRNAs in HNSCC and corresponding paracancerous tissues. A total of 1,053 differentially expressed mRNAs were identified, comprising 377 up-regulated and 676 down-regulated mRNAs with fold change > 2.0, p<0.05 (**Figure 1A**). The visualisation of the screened differentially expressed mRNAs was conducted using a volcano plot (**Figure 1B**). The microarray results indicated that 14-3-3ζ expression was elevated in HNSCC. To corroborate the findings as mentioned above, RT-PCR experiments were conducted, which suggested that the mRNA expression of 14-3-3ζ was markedly elevated in all three HNSCC cell lines relative to HOK (**Figure 1C**). In all five pairs of HNSCC and paracancerous tissue samples collected in this experiment, 14-3-3ζ was found to be highly expressed in HNSCC tissues compared with paracancerous tissues (**Figures 1D–G**).

### 14-3-3ζ is significantly associated with the development and prognosis of HNSCC

**Figure 2:**
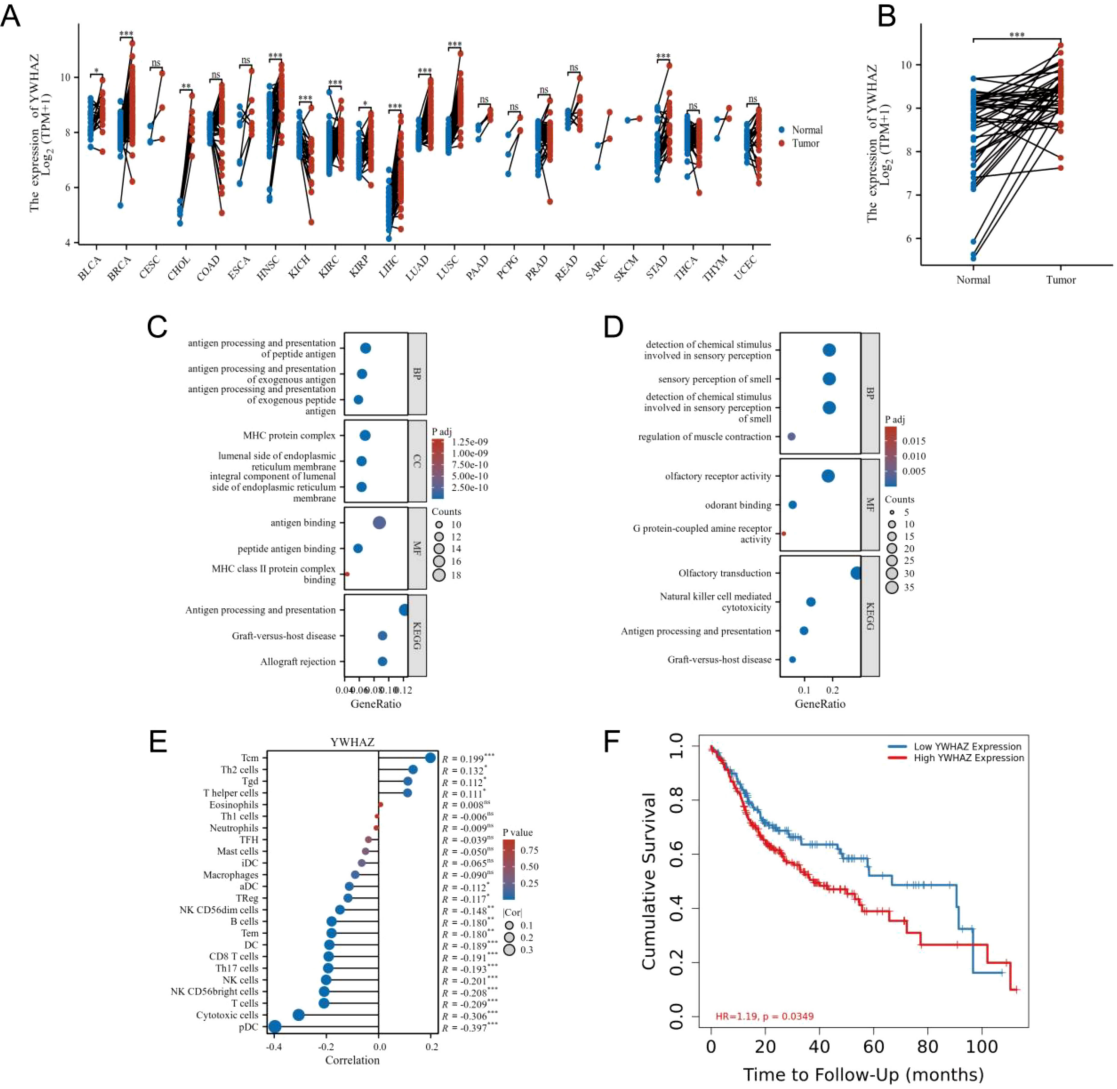
14-3-3ζ is significantly associated with the development and prognosis of HNSCC. **(A)** A pan-cancer analysis revealed that 14-3-3ζ expression was markedly elevated in 11 malignant tumours relative to normal tissues. **(B)** Data from the TCGA database indicated that 14-3-3ζ expression was relatively elevated in HNSCC. **(C, D)** The selected differential mRNAs were subjected to GO analysis and KEGG enrichment. **(E)** The infiltration of immune cells in HNSCC in the context of 14-3-3ζ. The relationship coefficient values are represented by dots, with the smallest values labelled from 0 to 0.1 and the most significant values labelled from 0.1 to 0.3. The p-value values are represented by a colour gradient, with the lowest p-values in blue and the highest p-values displayed in red. **(F)** Effect of 14-3-3ζ on the prognosis of HNSCC. Red represents a high expression of 14-3-3ζ, while green represents a low expression of 14-3-3ζ. (No significance(ns)>0.05, *p<0.05, **p<0.01, ***p<0.001, ****p<0.0001).

We analysed the different expressions of the 14-3-3ζ gene in pan-cancer in the GEPIA2 database. The results showed that the expression of 14-3-3ζ was significantly elevated in 11 malignant tumours compared with normal tissues (**Figure 2A**). Meanwhile, the data in the TCGA database showed that the expression of 14-3-3ζ was relatively high in HNSCC (**Figure 2B**). Immediately after that, we performed GO and KEGG analysis, and the results showed that the dysregulated mRNAs were enriched in biological processes (BPs) such as immune response and signalling, The most enriched molecular functions (MFs) were mainly related to receptor activity and receptor binding (**Figures 2C, D**). Among them, 14-3-3ζ may be involved in the BP through “protein targeting” (GO: 0006605), “anti-apoptosis” (GO: 0006916), and “signalling” (GO: 0006916), and “signalling” (GO: 0006917). By analysing the infiltration of 14-3-3ζ in 24 immune cells in HNSCC from the data downloaded from the TCGA database, we found that 14-3-3ζ was significantly correlated with 18 immune cells (**Figure 2E**, **Table 1**). We analysed the clinical data of 502 HNSCC patients from the TCGA database and found that the expression of 14-3-3ζ was significantly correlated with radiotherapy, histological grading, and anatomical tumour subdivision (**Table 2**). The same sample data were analysed for prognosis, and the overall survival time of the two groups showed that the overall survival time of the 14-3-3ζ high expression group was significantly lower than that of the low expression group (**Figure 2F**).

**Table 1:** Correlation between 14-3-3ζ and immune cells.

Immune cell	Correlation coefficient	P value
aDC	-0.108	0.015
B cells	-0.241	<0.001
CD8 T cells	-0.231	<0.001
Cytotoxic cells	-0.318	<0.001
DC	-0.102	0.022
Eosinophils	0.134	0.003
Neutrophils	0.123	0.006
NK CD56bright cells	-0.219	<0.001
NK CD56dim cells	-0.185	<0.001
NK cells	-0.172	<0.001
pDC	-0.353	<0.001
T cells	-0.227	<0.001
Tcm	0.157	<0.001
Tem	-0.208	<0.001
Tgd	0.159	<0.001
Th17 cells	-0.097	0.030
Th2 cells	0.134	0.003
TReg	-0.145	0.001

**Table 2:** Clinical molecular characteristics of high and low expression of 14-3-3ζ in HNSCC.

Clinical features	Case(n=502)	Low expression (%)	High expression (%)	P value
T-stage				0.8020
T1	44	24 (4.78%)	20 (3.98%)	
T2	144	72 (14.34%)	72 (14.34%)	
T3	131	68 (13.55%)	63 (12.55%)	
T4	183	87 (17.33%)	96 (19.13%)	
N-stage				0.6410
N0	239	120 (23.9%)	119 (23.71%)	
N1	96	46 (9.16%)	50 (9.96%)	
N2	160	83 (16.53%)	77 (15.34%)	
N3	7	2 (0.4%)	5 (1%)	
M-stage				0.2014
M0	492	244 (48.61%)	248 (49.4%)	
M1	10	7 (1.39%)	3 (0.6%)	
Radiotherapy				0.0127*
Yes	187	80 (15.94%)	107 (21.31%)	
No	315	171 (34.06%)	144 (28.69%)	
Histological grade				0.0061**
G1	62	29 (5.78%)	33 (6.56%)	
G2	310	141 (28.09%)	169 (33.66%)	
G3	127	78 (15.54%)	49 (9.77%)	
G4	3	3 (0.6%)	0 (0%)	
Anatomical tumour subdivision				< 0.0001****
Alveolar process	18	12 (2.4%)	6 (1.2%)	
Root of tongue	23	13 (2.6%)	10 (2%)	
Buccal mucosa	22	9 (1.8%)	13 (2.6%)	
Floor of mouth	61	26 (5.2%)	35 (7%)	
Palatum durum	7	0 (0%)	7 (1.4%)	
laryngopharynx	10	5 (1%)	5 (1%)	
throat	111	54 (10.8%)	57 (11.4%)	
lip	3	2 (0.4%)	1 (0.2%)	
Mouth cavity	72	34 (6.8%)	38 (7.6%)	
The tongue	126	55 (11%)	71 (14.1%)	
oropharynx	9	6 (1.2%)	3 (0.6%)	
tonsil	40	35 (7%)	5 (1%)	

(*p<0.05, **p<0.01, ****p<0.0001).

### Identification of circRNA_036186-miR-193b-3p-14-3-3ζ-regulatory axis

**Figure 3:**
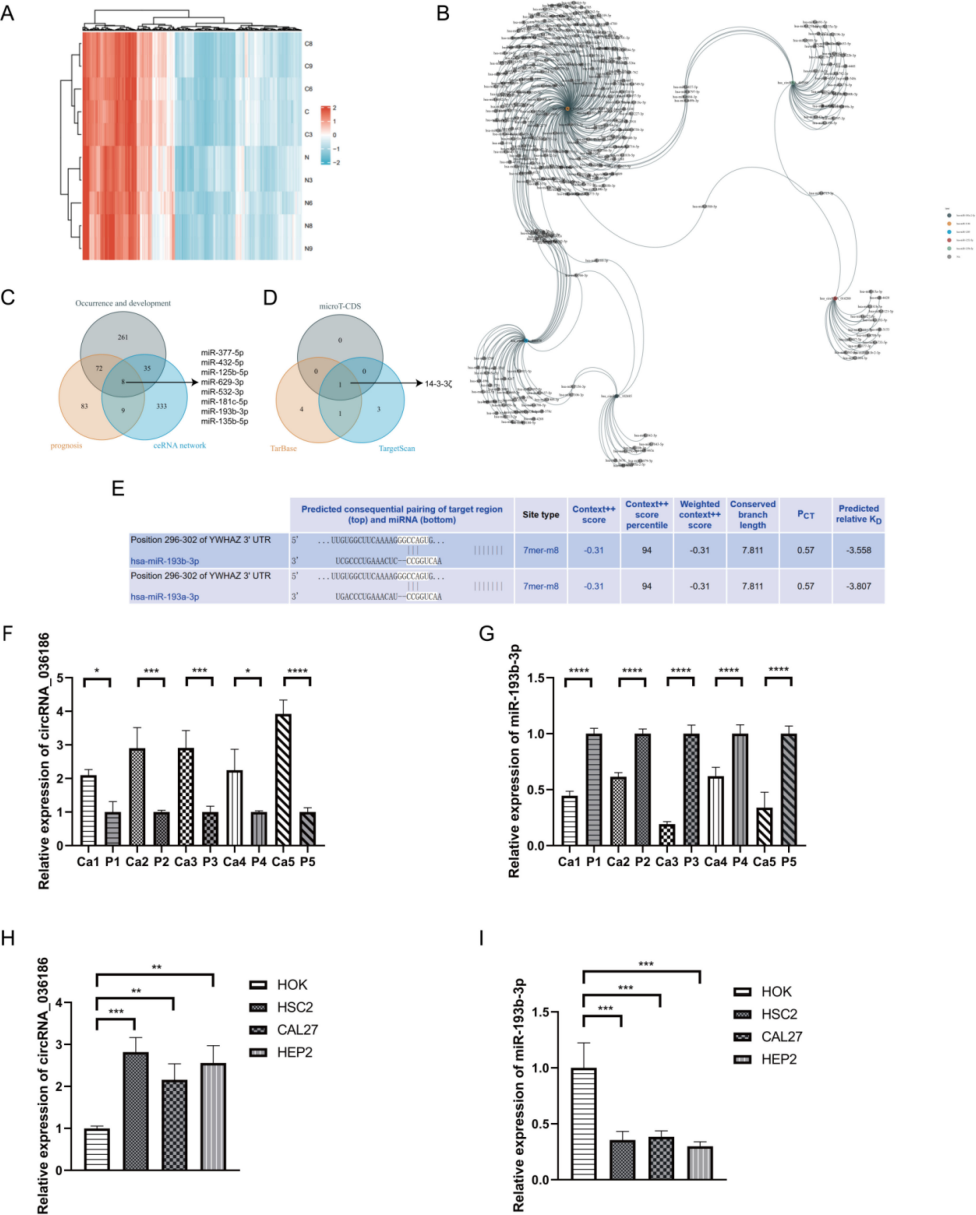
Identification of circRNA_036186-miR-193b-3p-14-3-3ζ-regulatory axis. **(A)** The heatmap illustrates the expression profiles of 287 circRNAs, of which 146 exhibited increased expression and 141 exhibited decreased expression. The colouration gradually transitions from blue to red, indicating increased expression. **(B)** The five most highly ranked circRNAs and their target miRNAs, each corresponding to a node, are shown with two interacting genes based on base sequence pairing connected by a solid line. **(C)** The intersection results of the HNSCC occurrence and development group, the prognosis group, and the competing endogenous RNA networks in OncomiR. **(D)** The predicted target mRNAs from three databases—Wayne**’**s map, miR-193b-3pmicroT-CDS, Tarbase, and TargetScan—were taken as intersections. **(E)** A search of the Tarbase database revealed that miR-193b-3p has a base sequence that binds and interacts with the 14-3-3ζ target. **(F, G)** The results of the RT-PCR experiments indicated that circRNA_036186 and miR-193b-3p exhibited elevated expression in HNSCC tissues relative to paracancerous tissues in five pairs of HNSCC and paracancerous tissue samples. **(H, I)** The results of RT-PCR experiments indicated that the mRNA expression of circRNA_036186 and miR-193b-3p was markedly elevated in all three HNSCC cell lines relative to HOK. (n=3, *p<0.05, **p<0.01, ***p<0.001, ****p<0.0001).

A high-throughput microarray analysis identified 12,366 circRNAs in HNSCC and corresponding paracancerous tissues. A total of 287 circRNAs were identified as exhibiting dysregulated expression. Of these, 146 displayed upregulated expression, and 141 displayed downregulated expression with fold change>1.5, *p*<0.05 (**Figure 3A**). The five most differentially expressed circRNAs were selected from the microarray data, and potential miRNA response elements were identified using TargetScan and miRanda software on both circRNA and mRNA sequences (**Table 3**). A competitive endogenous RNA set was generated by merging with common targeting miRNAs, comprising five circRNAs, 385 miRNAs, and 5148 mRNAs. To further investigate the interactions of these competing endogenous RNAs in HNSCC tissues, we used only differentially expressed mRNAs from the mRNA microarray data and constructed an HNSCC-specific competitive endogenous RNA network comprising five circRNAs and 385 miRNAs (**Figure 3B**). The OncomiR software revealed that 376 of the miRNAs were related to tumorigenesis and development. In contrast, 172 were related to tumor prognosis in HNSCC. The intersection of the two sets yielded 80 miRNAs that were present in both. Finally, we took the intersection of the miRNAs in the competitive endogenous RNA network with the 80 miRNAs mentioned above, and the results showed that eight miRNAs were present in both (**Figure 3C**), in which circRNA_014280 did not target the eight miRNAs discussed above. The results from TargetScan, Diana-TarBase, and Diana-microT-CDS were taken as the intersection, and as a result, only miR-193b-3p matched with 14-3-3ζ for target binding (**Figures 3D, E**). To corroborate the findings above, we conducted RT-PCR experiments, which indicated that the expression of circRNA_036186 was markedly elevated in HOK intersecting HNSCC cell lines. In contrast, the expression of miR-193b-3p was significantly diminished compared to HOK (**Figures 3F, G**). In the five pairs of HNSCC and paracancerous tissue samples collected in this experiment, circRNA_0361863p was highly expressed in HNSCC tissues compared with paracancerous tissues. In contrast, miR-193b-3p was lowly expressed in HNSCC tissues (**Figures 3H, I**).

**Table 3:** Top 5 circRNA.

circRNA	Express	Fold Change	P-value	FDR	CircRNA Typ
has_circRNA_014280	Up	4.005569	0.0000401	0.017333277	Exon
has_circRNA_402089	Up	3.2867219	0.0000464	0.018507674	Exon
has_circRNA_036186	Up	3.2503065	0.0000705	0.022943867	Exon
has_circRNA_404474	Up	3.1449976	0.0000822	0.024789243	Exon
has_circRNA_102485	Down	4.3172347	0.0000171	0.009591013	Exon

### Effect of down-regulation of circRNA_0361863p and miR-193b-3p on 14-3-3ζ in HSC2

**Figure 4:**
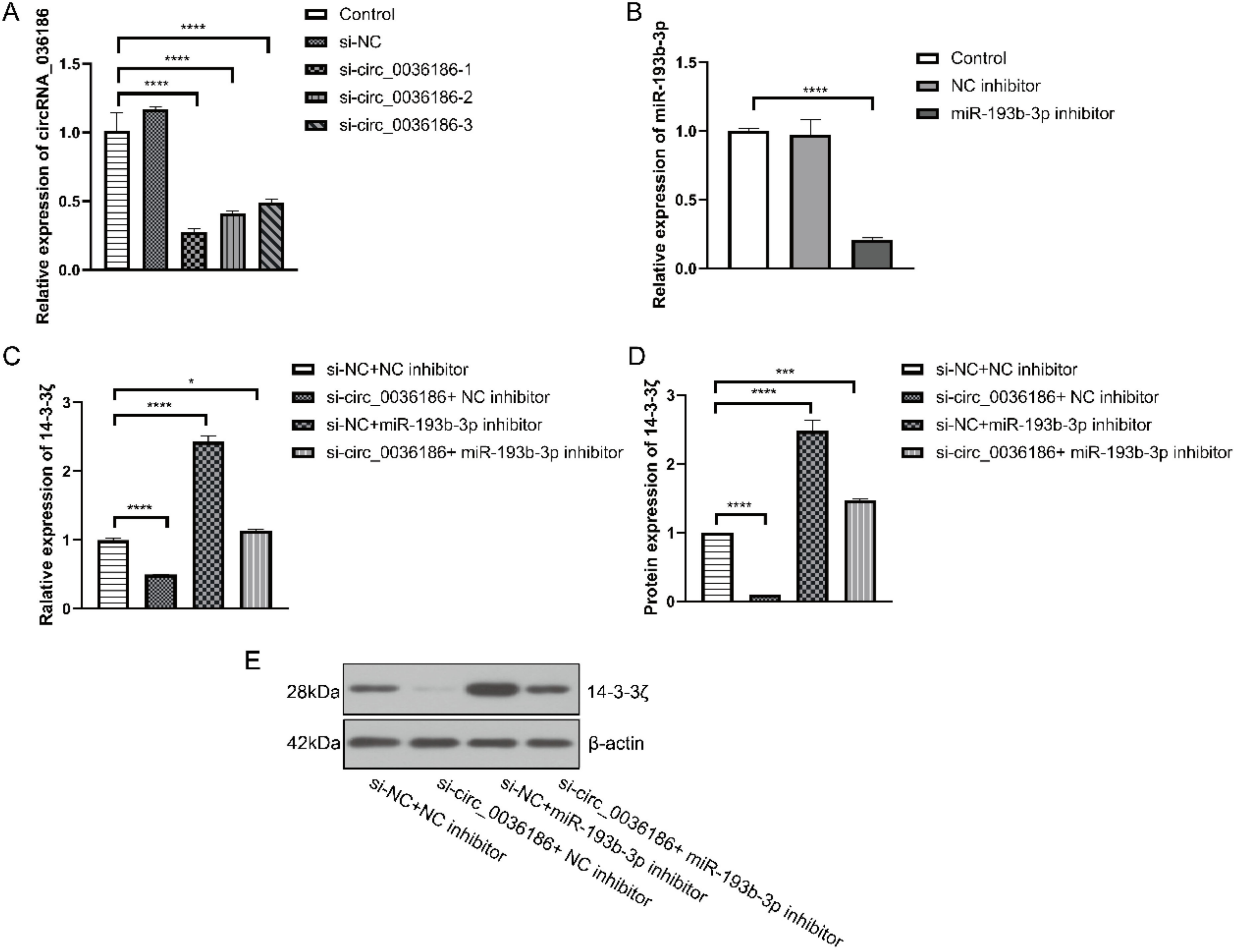
Effect of down-regulation of circRNA_0361863p and miR-193b-3p on 14-3-3ζ in HSC2. **(A)** The results of the RT-PCR experiment indicated that si-circ_0036186 had been successfully transfected into HSC2. **(B)** The results of the RT-PCR experiments indicated that miR-193b-3p had been successfully transfected into HSC2. **(C)** Reverse transcription polymerase chain reaction (RT-PCR) experiments were conducted to ascertain the impact of transfection on 14-3-3ζ mRNA expression. **(D, E)** Western blotting experiments were conducted to ascertain the impact of transfection on 14-3-3ζ protein expression. (n=3, *p<0.05, ***p<0.001, ****p<0.0001).

Three groups of si-circ_0036186 were successfully transfected into HSC2 cells. The RT-PCR results demonstrated a notable decline in the expression of circ_0036186 in HSC2 following the transfection of all three groups of si-circ_0036186. Among the three groups of si-circ_0036186, si-circ_0036186-1 demonstrated the most effective interference and was therefore selected for subsequent experiments (**Figure 4A**). The miR-193b-3p inhibitor was transfected into HSC2 cells, resulting in a significant reduction of miR-193b-3p expression, as confirmed by RT-PCR analysis (**Figure 4B**).

The RT-PCR and Western blotting results indicate a significant decrease in the expression level of 14-3-3ζ in HSC2 in group B. In contrast, the expression level of 14-3-3ζ was significantly increased in HSC2 in group C. Additionally, the expression level of 14-3-3ζ was increased in HSC2 in group D but relatively decreased compared to group C (**Figures 4C–E**).

### Effect of down-regulation of circRNA_0361863p and miR-193b-3p on the proliferation, migration, invasion, and scratch healing rate of HSC2 cells

**Figure 5:**
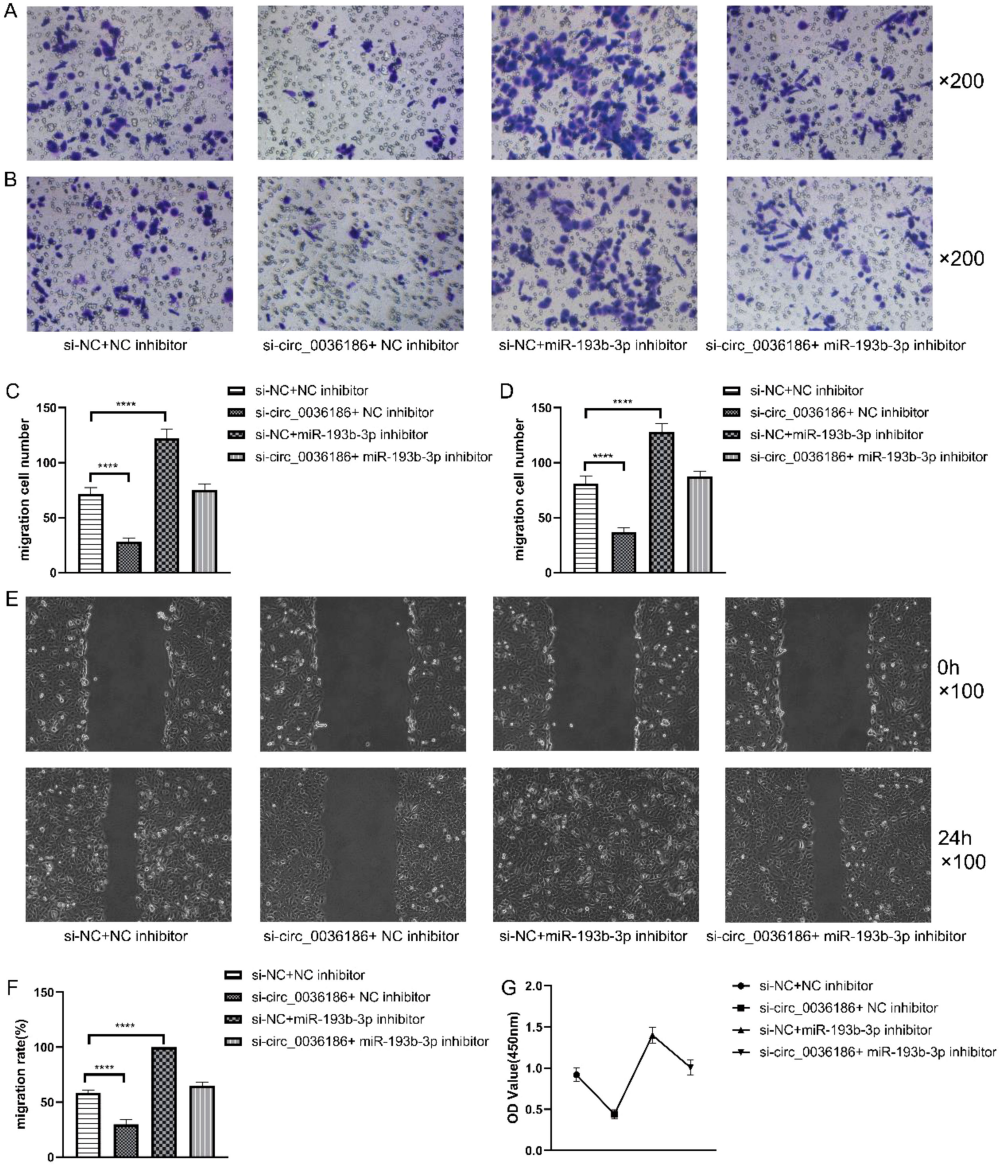
Effect of down-regulation of circRNA_0361863p and miR-193b-3p on the proliferation, migration, invasion, and scratch healing rate of HSC2 cells. **(A–D)** The Transwell assay assessed the impact of circRNA_0361863p and miR-193b-3p down-regulation on cell migration and invasion of HSC2. **(E, F)** The Scratch test assessed the scratch healing rate of HSC2 after the down-regulation of circRNA_0361863p and miR-193b-3p. **(G)** The cell activity of HSC2 was evaluated after the down-regulation of circRNA_0361863p and miR-193b-3p through CCK-8 experiments. (n=3, ****p<0.0001).

The transwell assay results demonstrated that the migration and invasion capacity of HSC2 was markedly diminished following the silencing of circ_0036186. In contrast, the migration and invasion capacity of HSC2 was significantly enhanced after the inhibition of miR-193b-3p expression in HSC2. When both were inhibited simultaneously, an increase in migratory and invasive abilities was observed in HSC2. Nevertheless, the migratory and invasive capabilities of the cells in Group D exhibited a comparatively diminished extent relative to those observed in Group B (**Figures 5A–D**). The scratch test yielded analogous results (**Figures 5E, F**).

The CCK-8 experiments demonstrated that the proliferation capacity of HSC2 was markedly diminished in group B, whereas it was enhanced significantly in group C. Group D exhibited an elevated proliferation capacity, albeit to a lesser extent than that observed in Group C (**Figure 5G**).

## Discussion

HNSCC is a highly heterogeneous, aggressive, and genetically complex malignancy that can arise in several locations (11). 14-3-3ζ plays a pivotal role in the processes of tumorigenesis and development and is involved in many signalling pathways (12–14). 14-3-3ζ is regulated by either miRNA or lncRNA and performs its functions by targeting downstream molecules, including protein kinases, apoptotic proteins, and metastasis-related molecules (15–17). In the present study, we sought to ascertain whether 14-3-3ζ expression was elevated in various malignant tumours and found that the expression 14-3-3ζ was markedly elevated in HNSCC cell lines and tissues. Our findings indicate that the overall survival time of individuals with high 14-3-3ζ expression is significantly reduced, and this is closely correlated with radiotherapy and histological grading. Therefore, it is proposed that the aberrant expression of 14-3-3ζ in HNSCC may serve as a potential biomarker for cancer diagnosis and chemotherapy drug resistance.

In recent years, an increasing number of experimental and clinical studies have demonstrated the significant role of ncRNAs in the pathophysiology and development of HNSCC (18). Among the various classes of ncRNAs, miRNAs are currently the most intensively studied. They can block protein translation at the transcriptional level or regulate the stability of their downstream target mRNAs by binding to MREs. In contrast to miRNAs, the functions and regulatory mechanisms of circRNAs are not yet well understood. However, it is known that circRNAs have three main tasks: uptake of miRNA sponges, binding of RNA-binding proteins, and translation of peptides (19).

An increasing number of studies have demonstrated that circular RNAs (circRNAs) regulate the expression of tumour-associated genes, primarily through the circRNA-microRNA-messenger RNA (mRNA) regulatory axis (8, 20, 21). The study revealed that many dysregulated mRNAs and circRNAs were present in HNSCC, which predominantly regulated biological processes such as immune response and signal transduction. Among the aberrantly expressed mRNAs and circRNAs, the expression levels of circRNA_036186 and 14-3-3ζ were significantly elevated in HNSCC samples compared to paraneoplastic tissues. By mapping the endogenous competing RNA network, we were able to identify a high degree of correlation between circRNA_036186, miR-193b-3p, and 14-3-3ζ, which suggests the potential involvement of this axis in regulating the development and prognosis of HNSCC.

To further determine the relationship between the three, we knocked down the expression of circRNA_036186 and miR-193b-3p in HSC2 *in vitro*. The study revealed that the down-regulation of circRNA_036186 resulted in a decrease in the expression of 14-3-3ζ in HSC2, which in turn led to a reduction in the proliferation, migration, and invasion abilities, as well as the scratch healing rate, of HSC2. In contrast, the down-regulation of miR-193b-3p increased the expression of 14-3-3ζ in HSC2, leading to enhanced proliferation, migration, and invasion ability and a higher scratch healing rate. Concurrent down-regulation of circRNA_036186 and miR-193b-3p expression increased 14-3-3ζ expression in HSC2, resulting in enhanced proliferation, migration, invasion ability, and scratch healing rate. These findings indicate that circRNA_036186 exerts a positive regulatory influence on 14-3-3ζ, whereas miR-193b-3p exerts a negative regulatory effect on 14-3-3ζ.

In light of the findings above, we have concluded that the circRNA_036186-miR-193b-3p-14-3-3ζ pathway may considerably influence the development and prognosis of various cancers, including human head and neck squamous cell carcinoma. It is regrettable that, to date, no studies have reported the function and mechanism of the circRNA_036186-miR-193b-3p-14-3-3ζ pathway in patients with head and neck squamous cell carcinoma (HNSCC). When considered alongside existing research data and the findings of this study, the novel endogenous circRNA molecule, circRNA_036186, may play a pivotal role in regulating 14-3-3ζ by suppressing the function of miR-193b-3p as a sponge, thus influencing the occurrence and progression of HNSCC.

### Limitations of the study

The subsequent phase of the study will involve the collection of further samples to give more insight into the expression of miR-193b-5p, circRNA_036186 and 14-3-3ζ in HNSCC. Moreover, as the present study offers only a preliminary analysis of the dysregulated mRNAs pathway in HNSCC through GO and KEGG analysis, subsequent research will refine the cellular experiments, add luciferase experiments and other basic experiments, in addition to constructing animal models to validate the circRNA_036186/miR-193b-5p/14-3-3ζ signalling pathway in HNSCC *in vivo*.

## Conclusion

we employed chip technology to ascertain circRNA, miRNA, and mRNA expression profiles in five pairs of HNSCC cancer tissues and their corresponding paracancerous tissues. Following cross-referencing the results with TCGA and other databases, circRNA_036186 was identified as a potential diagnostic marker and therapeutic target for HNSCC. Furthermore, this is the inaugural proposal of the impact of the circRNA_036186-miR-193b-3p-14-3-3ζ pathway on the progression and prognosis of HNSCC. Also, it establishes a foundation for investigating the role of circRNA in HNSCC.

## Materials and methods

### Acquisition of HNSCC samples

During the Oral and Maxillofacial Surgery at Binzhou Medical University, five samples of 3mm*3mm*3mm carcinoma and corresponding paracancerous tissues were taken, and five pairs of cancer and corresponding paracancerous tissues were selected from China Medical University (**Table 4**). The samples were sent to Kangcheng Biological Company with dry ice for total RNA sample detection, library construction, library detection, and computer sequencing. The Ethics Committee of Binzhou Medical University Affiliated Hospital approved all experimental procedures and programs.

**Table 4:** Clinical information of the five sample patients tested by microarray.

Patient number	Sex	Age	Position	Pathological diagnosis	Histological classification	TNM
1	Male	53	Abdomen of tongue, Floor of mouth	HNSCC	G2	T2N0M0
2	Male	33	Abdomen of tongue	HNSCC	G2	T2N0M0
3	Female	63	Lateramargin of tongue	HNSCC	G2	T2N1M0
4	Male	59	Lateral margin of tongue	HNSCC	G2	T2N1M0
5	Male	43	Lateral margin of tongue	HNSCC	G1-G2	T2N1M0

Inclusion criteria: (1) The diagnosis of HNSCC was made by the Department of Pathology of the Affiliated Hospital of Binzhou Medical College or the Department of Pathology of the Affiliated Hospital of China Medical University. (2) It was the first surgery without radiotherapy and chemotherapy. (3) All patients were informed and consented to participate. The ethics of this study were approved by the Affiliated Hospital of Binzhou Medical College and the Affiliated Stomatological Hospital of China Medical University (No. KYLL-2022-117).

### Cell lines and cultures

HNSCC cell lines (HSC2, CAL27, HEP2) and Human oral mucosal keratinocytes (HOK) were obtained from Cobioer (Nanjing, China) and cultured in DMEM supplemented with 10% fetal bovine serum, 100 U/mL penicillin, and 100 µg/mL streptomycin.

### Microarray analysis

RNase R is employed to eliminate linear RNA and increase the concentration of circRNA. We used the Arratstar Super RNA Labeling Kit to amplify the enriched circRNA, which was then transcribed into fluorescent cRNA.The labelled cRNA was hybridised to the Arraystar Human circRNA V2 chip. Subsequently, the glass slides were cleaned, and the array was scanned utilising an Agilent G2505C scanner. The array images were analysed using the Agilent Feature Extraction software, while the data was processed with the R software package.

### Analysis of differential genes and functional enrichment

We used edgeR to analyse the differences in high-throughput sequencing data and then used the adjusted p-value to determine the significance level. We applied a *p*-value<0.05 for differential filtering and identified mRNA, miRNA, and circRNA exhibiting considerable expression differences.

GOseq and KOBAS software further analysed the differential expression of mRNA in sequencing, and the analysis results were visualised.

### Survival curve and immune infiltration analysis

We obtained information on HNSCC patients from the TCGA database and divided HNSCC patients into 14-3-3ζ high expression group and 14-3-3ζ low expression group. Next, we utilised R software to scrutinise the OS of 14-3-3ζ in HNSCC patients.

We examined the immune infiltration through the ssGSEA method and used the log-rank test to determine whether there was a statistical difference between the two groups.

### Establishment of a competitive endogenous RNA network

We predict miRNA binding sites in circRNA sequences using TargetScan and miRanda prediction software.

Our parameter settings are miRNA coverage≥0.1, context+<-4.999999977648258e-2; commonNum≥1; ceRNA type=protein coding; structure>140, *p<*0.05; contexe<-4.999999977648258e-2; energy <-10. We then plotted the competitive endogenous RNA network using Cytoscape softwar**e.**

### Screening of miRNA related to tumour development and patient survival

We employed the OncomiR online software (http://www.oncomir.org/) to identify miRNA associated with tumourigenesis and survival in HNSCC patients.

### Prediction of targeted binding of miRNA-mRNA

TargetScan, Diana-microT-CDS, and Diana-TarBase software forecast the associated mRNA targets of miRNA, while Diana-miRPath scrutinises miRNA-related pathways.

### Verification of circRNA_036186, miR-193b-5p and 14-3-3ζ expression in HNSCC

We utilised RT-PCR and Western Blot techniques to confirm the expression of circRNA_036186, miR-193b-5p, and 14-3-3ζ in five HNSCC and corresponding paracancerous tissue. The primer sequences are listed below hsa_circ_0036186 F: ATAGAGCCTACCTGTATGTCA, hsa_circ_0036186 R: GAGAAGTTCAGACGAGCC, 14-3-3ζ F: AGGCTGAGCGATATGAT, 14-3-3ζ R: TCCAAGATGACCTACGG, β-actin F: GGCACCCAGCACAATGAA, β-actin R: TAGAAGCATTTGCGGTGG, has-miR-193b-3p F: AACTGGCCCTCAAAGTCCCGCT, U6 F: GGAACGATACAGAGAAGATTAGC, U6 R: TGGAACGCTTCACGAATTTGCG.

### Constructing and transfecting siRNAs and inhibitor

Three siRNAs were designed for hsa_circ_0036186 and transfected into HSC2 cells (hsa_circ_0036186 si-1: AGCUGAAGCACCGCCCAGCUUTT AAGCUGGGCGGUGCUUCAGCUTT, hsa_circ_0036186 si-2: AGCACCGCCCAGCUUCCCGAUTT AUCGGGAAGCUGGGCGGUGCUTT, hsa_circ_0036186 si-3: AAGCACCGCCCAGCUUCCCGATT UCGGGAAGCUGGGCGGUGCUUTT, hsa_siRNA_NC: UUCUCCGAACGUGUCACGUTT ACGUGACACGUUCGGAGAATT). The expression level of cellular hsa_circ_0036186 was detected by RT-PCR 48 hours after transfection. We designed and synthesised an inhibitor for hsa_miR-193b-3p, which was then transfected into HSC2 cells (hsa_miR-193b-3p inhibitor: AGCGGGACUUUGAGGGCCAGUU, hsa_inhibitor NC: UUGUACUACACAAAAGUACUG).

The expression level of cellular hsa_miR-193b-3p was detected 48 hours after transfection using RT-PCR. Subsequently, HSC2 cells were divided into four groups for further experiments: A) si-NC + NC inhibitor, B) si-NC + miR-193b-3p inhibitor, C) si-circ_0036186 + NC inhibitor, and D) si-circ_0036186 + miR-193b-3p inhibitor.

### Validation of 14-3-3ζ expression levels after transfection

RT-PCR and Western blotting verified the expression of 14-3-3ζ mRNAs and proteins in the four cell groups listed above.

### Verification of HSC2 cell activity, proliferation, migration, and invasion ability after transfection

The activity of HSC2 cells was detected using the CCK-8 assay 48 hours post-transfection. The proliferation ability of HSC2 cells was detected using the scratch assay 24 hours post-transfection. The ability of HSC2 invasion and migration was detected using the Transwell assay after transfection.

### Statistical analysis

Statistical analysis was conducted using GraphPad Prism version 10.0. The data are presented as the mean value accompanied by the standard deviation. Data exhibiting normal distribution were compared using ordinary multivariate ANOVA with repeated measures. The differential expression of mRNAs and circRNAs was investigated using edge R software to organise the microarray data. The differential size was determined by fold change (FC) multiplicity; p-value and |FC| were used to screen differential genes, and FC represents tumour vs. non-tumour. Survival analysis was performed using R software to analyse the OS of 14-3-3ζ in HNSCC patients, and we used the log-rank test to compare whether there was a statistical difference between the two groups.

## Data Availability

The data supporting this study's findings are available from the corresponding author upon reasonable request.
